# Divergent Photoperiodic Responses in Hypothalamic *Dio3* Expression and Gonadal Activity Between Offspring and Paternal Brandt’s Voles

**DOI:** 10.3390/ani15040469

**Published:** 2025-02-07

**Authors:** Lewen Wang, Zhengguang Li, Ying Song, Ning Li, Xiao-Hui Liu, Dawei Wang

**Affiliations:** 1 State Key Laboratory for Biology of Plant Diseases and Insect Pests, Institute of Plant Protection, Chinese Academy of Agricultural Sciences, Beijing 100193, China; happywwener@163.com (L.W.); 15515629104@163.com (Z.L.); songying01@caas.cn (Y.S.); liuxiaohui@caas.cn (X.-H.L.); 2 Western Agricultural Research Center, Chinese Academy of Agricultural Sciences, Changji 831100, China; 3 Key Laboratory of Biohazard Monitoring and Green Prevention and Control in Artificial Grassland, Ministry of Agriculture and Rural Affairs, Institute of Grassland Research, Chinese Academy of Agricultural Sciences, Hohhot 010010, China

**Keywords:** hypothalamic *Dio3*, gonadal activity, age differentiation, changing photoperiod, Brandt’s vole

## Abstract

The postnatal development of gonadal glands in seasonal breeders, particularly small rodent species, is influenced by photoperiodic patterns. However, little research has been conducted on the effects of pattern similarity and age differentiation especially in molecular features. This study compares the postnatal development of gonadal glands and the expression of hypothalamic genes related to reproductive regulation in male offspring of Brandt’s voles (*Lasiopodomys brandtii*) born under three types of changing photoperiodic patterns: increasing long photoperiod (ILP, 12 h + 3 min/day), natural increasing long photoperiods (NLPs), and decreasing short photoperiods (DSPs, 12 h − 3 min/day), as well as in their paternal voles exposed to these patterns at the same period. Results indicate that over the course of 12 postnatal weeks, gonadal development, including organ masses and serum testosterone levels, exhibited similar profiles between the ILP and NLP groups, which were significantly higher than those observed in DSP offspring. Hypothalamic type 3 iodothyronine deiodinase (*Dio3*) exhibited significantly higher expression in the DSP group from postnatal week 4 to 8 compared to the other two groups. These physiological and molecular differences gradually decreased with age in offspring, but were never observed in the paternal voles, indicating divergent photoperiodic responses between the two ages. The synchronous profiles observed between hypothalamic *Dio3* expression and gonadal activities underscore its crucial role in interpreting photoperiodic signals and regulating gonadal development in Brandt’s voles.

## Simple Summary

This study investigated an age-dependent photoperiod sensitivity mechanism in male Brandt’s voles, a small rodent species. We examined the gonadal development and expression profiles of reproductive-related genes in the hypothalamus of male offspring born under three different photoperiod conditions, as well as in their fathers. The results showed that both artificial and natural increasing long photoperiod treatments led to similar phenotypes and gene expression characteristics in male offspring, specifically a significantly higher level of gonadal development and a notably lower expression of hypothalamic *Dio3* compared to the decreasing short photoperiod group. However, paternal voles did not exhibit any significant response to the applied photoperiod. These results suggest divergent photoperiodic responses between the two ages and highlight the crucial role of hypothalamic *Dio3* in interpreting photoperiodic signals and regulating gonadal development in Brandt’s voles.

## 1. Introduction

Seasonal breeding is an adaptive strategy observed in over 4000 mammal species, especially those inhabiting middle and high-latitude regions [1]. These animals have evolved the ability to synchronize their reproductive physiology with the external annual day length cycle, known as the photoperiod, through internal seasonal clocks [2,3]. Adult animals undergo spontaneous activation and regression of their gonadal glands in the breeding and non-breeding seasons, respectively [4], a process that can also be influenced by artificially manipulated photoperiods, such as stable or gradually changing photoperiods [5,6]. However, the regulation of reproductive development in juvenile animals, particularly in some small rodent species, is more complex. For instance, newborns may need to accelerate the maturation of their gonadal glands within a few weeks if born in the early period of the breeding season, or delay puberty until the following spring if born in the late stage of the breeding season [7]. The mechanisms governing these postnatal developmental trajectories remain largely unknown.

Developmental plasticity reflects genetic variability influenced by early-life events, such as the prenatal and early postnatal periods [8]. Research has indicated that in certain hamster and vole species, the fetus can perceive seasonal photoperiodic cues from maternal melatonin in uterus [9,10]. Melatonin, secreted by the pineal gland during the night, conveys information about seasonal day length, coordinating gonadal activity with seasonal shifts in adult mammals. Previous studies have shown that pineal gland removal prevents gonadal atrophy in hamsters exposed to short photoperiods (SPs, day length < 12 h/day), while exogenous melatonin inhibits gonadal function under long photoperiods (LPs, day length > 12 h/day) [11]. These seasonal signals of the photoperiod and melatonin can be transmitted to fetuses during pregnancy or to juveniles during lactation through their dams. For example, an intermediate photoperiod (14 h) suppressed testicular development in juveniles born under LP (16 h), while promoting testicular growth in those born under SP (8 h) by the age of 50 days [10]. Furthermore, administering melatonin to dams from pregnancy through lactation reduced the testicular weight of juveniles by day 28 [9]. Despite the clear transmission of photoperiod signals from mother to offspring, the molecular mechanisms underlying the response to the photoperiod and its regulation of gonadal development in juveniles remain unknown.

The Pars tuberalis (PT) in the pituitary gland and tanycytes in the hypothalamus are recognized as key regulators of seasonal changes. PT cells, rich in melatonin receptor 1 (MT1), secrete thyroid-stimulating hormone (TSH) in adult rodents [12,13,14]. The expression of TSHβ, the beta subunit of TSH, is influenced by the duration of melatonin secretion, with high expression under LP conditions and low expression under SP exposure [15,16,17]. TSH binds to receptors on tanycytes in the third ventricle, regulating local triiodothyronine (T_3_) levels by modulating iodothyronine deiodinases, specifically type 2 (DIO2) and type 3 (DIO3), which respectively activate and deactivate T_3_ [18]. Research conducted in both an artificial setting [6,14,19,20] and the natural environment [21] shows that LP conditions upregulate *Dio2* and downregulate *Dio3* in the rodent hypothalamus, while SP conditions reverse this pattern. Hypothalamic *Dio3* exhibits a more pronounced response to changes in the photoperiod compared to *Dio2* across different species, suggesting its conserved role in seasonal adaptations [6,19,22]. Additionally, neuropeptides Kisspeptin (encoded by *Kiss1*) and RFamide-related peptide 3 (encoded by *Rfrp3*, also known as gonadotropin inhibitory hormone, GnIH) modulate gonadotropin-releasing hormone (GnRH) neuronal activity in rodents [23,24,25,26]. These peptides are thought to connect the TSH-deiodinase pathway with the hypothalamic–pituitary–gonadal (HPG) axis, exhibiting a significant response to photoperiod variations, thyroid hormones, and gonadal hormones [27,28]. Existing literature has predominantly focused on adult rodents, with a few exceptions [22,29,30].

Juvenile rodents may not consistently exhibit the same photoperiodic response as adults. Adult Siberian (*Phodopus sungorus*) and Syrian hamsters (*Mesocricetus auratus*) exhibited significant reproductive inhibition under SP compared to LP conditions, as did juvenile Siberian hamsters [31,32]. However, juvenile Syrian hamsters did not show the same reproductive inhibition under SP conditions [33]. Turkish hamsters (*Mesocricetus brandti*) demonstrated a photoperiodic response similar to Siberian hamsters [34], while Djungarian dwarf hamsters (*Phodopus campbelli*) only partially suppressed reproductive functions under SP conditions [35]. The molecular mechanisms underlying these photoperiodic responses in juvenile rodents, and their distinctions from those of adults, remain unclear.

Brandt’s voles (*Lasiopodomys brandtii*), a small herbivorous rodent species with a lifespan of less than 14 months and no hibernation habits, inhabit the steppes of the Mongolian plateau in China, the Republic of Mongolia, and the Baikal Lake region of Russia [36,37,38,39,40]. They breed exclusively from spring (early March) to autumn (late August) in their natural environment [36,37,41,42]. Male voles experience a significant reduction in testes mass of 20–100 times during the non-breeding season compared to the breeding season [21,43]. However, gonadal activity does not synchronize across different age groups: adult males maintain functional testicular activity until late summer, while most newborn males delay puberty until the following spring, except for a few born early in spring [44]. Overwintered males, the primary contributors to the breeding season, do not survive the second winter [21,43]. Previous studies have shown synchronous seasonal changes between photoperiodic genes (*Dio2*&*3*) in the hypothalamus with the photoperiod and testes mass in wild voles [21]. Additionally, the higher expression of hypothalamic *Dio3* occurs under decreasing SP conditions following inhibited gonadal development compared to increasing LP exposure [45]. However, it is still unclear whether adult male voles respond to photoperiodic changes in the same way as juveniles, and the exact role of hypothalamic genes in this process remains to be elucidated.

In this study, we hypothesized that the photoperiod is the primary factor influencing the seasonal breeding of Brandt’s voles, affecting both somatic and gonadal development in offspring born under LP and SP conditions, and leading to distinct expression patterns of major genes regulating seasonal breeding in the hypothalamus. Additionally, we predicted variations in photoperiodic responses between adult and juvenile voles. To test these hypotheses, voles were exposed to three photoperiodic patterns: two groups subjected to 12 h + 3 min/day and 12 h − 3 min/day, as outlined in Qiao et al. (2024), and one group exposed to the natural spring sinusoidal photoperiod in Beijing (~40° N). The 3 min daily change aligns with the maximum rate observed in the annual photoperiodic cycle in Inner Mongolia (~45° N). Gonadal development and hypothalamic gene expression were compared in offspring and paternal voles exposed to the three photoperiodic treatments to investigate (1) similarities in gonadal development and hypothalamic gene expression in offspring under comparable photoperiodic patterns; (2) potential differential photoperiodic responses between older and younger voles; and (3) synchronous changes in key hypothalamic genes with gonadal development, particularly regarding the seasonal breeding of Brandt’s voles.

## 2. Materials and Methods

### 2.1. Animals and Housing Conditions

Voles have been selectively bred in our laboratory in Beijing (40°1′ N, 116°17′ E) for over 20 generations since 2007. They are housed in plastic cages (29 × 17.5 × 13 cm) containing wood shavings, with 3–5 voles per cage to encourage social interaction. The holding room is kept at a constant temperature of approximately 20 °C and follows a natural photoperiod. The bedding material is replaced weekly to uphold cleanliness and minimize stress. Voles have ad libitum access to standard rabbit chow and purified water. Daily health checks are conducted, and any signs of distress or illness are promptly addressed. All procedures follow the institutional guidelines for animal use and care of the Institute of Plant Protection at the Chinese Academy of Agricultural Sciences (Protocol No. Ipp-201606R010).

### 2.2. Experiment Design

**Figure 1:**
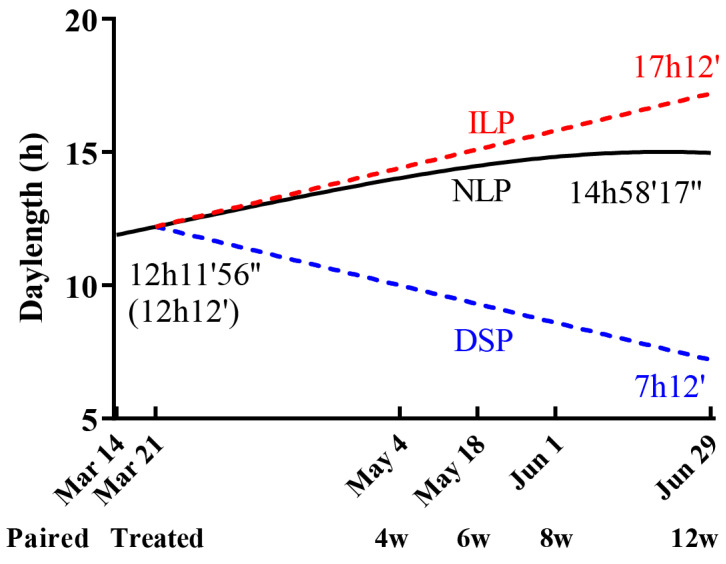
Photoperiod paradigm. The horizontal axis shows the critical timepoints, including paired, treated, and sampling, while the line represents the day length of three photoperiod treatments. Solid and dotted lines differentiate between natural and simulated photoperiods, with red indicating increasing and blue indicating decreasing day lengths. The initial natural day lengths are denoted above the simulated timings in brackets on the left, while the terminal day lengths are indicated at the right end of the lines. ILP: increasing long photoperiod; DSP: declining short photoperiod; NLP: natural spring long photoperiod; w: postnatal weeks.

To detect the effects of photoperiod on juvenile and paternal voles, pregnant female voles and their male counterparts were subjected to varying day lengths. Around 6-month-old voles from our laboratory breeding colony, raised under natural photoperiod conditions, were randomly selected. A total of 60 parent pairs were bred one week prior to the spring equinox in Beijing (11 h 53′27″, 7 March) under natural photoperiod conditions. After a week of pairing, the parent pairs were individually housed and divided into three groups for varied photoperiod treatments. One group was maintained under a natural increasing long photoperiod (NLP, with day length starting from 12 h 12′ on 21 March). The other two groups experienced a daily increase or decrease of 3 min in day length starting from the same day, labeled as the increasing long photoperiod (ILP) and decreasing short photoperiod (DSP) groups, respectively. The 3 min interval corresponded to the maximum annual day length change in the voles’ natural habitat (3′15″). The lighting-up time was set at 6:15 a.m. to coincide with the local sunrise. For simplicity, adjustments were made only to the daily turn-off time (See Figure 1). The NLP group was housed in rooms with transparent windows to allow natural light exposure, ensuring a photoperiod that closely mirrored external sunlight. In contrast, the ILP and DSP groups were kept in completely dark rooms, with lighting controlled by automatic timers and no external light sources.

After being exposed to three different photoperiod treatments, offspring were born about two weeks later, with each group consisting of 14 to 18 cohorts. The average litter size was consistent across groups (6.5–6.6), with litter sizes ranging from 3 to 11, 3 to 9, and 4 to 10 in the NLP, ILP, and DSP groups, respectively. To minimize litter effects, male offspring from the same litter were randomly allocated to different timepoints for sampling at postnatal week 4, 6, 8, and 12. This approach generally ensured that male offspring sacrificed at the same timepoint originated from different litters. To avoid disturbing the lactating females, which could lead to infanticide and compromise subsequent sampling, sampling points were set after the offspring were weaned at postnatal week 4. Due to the limited sampling size (18–20 in each group), paternal voles were sampled only at three of these timepoints (postnatal week 4, 8, 12). The specific sampling numbers for each group at each timepoint are detailed in Table 1.

### 2.3. Hypothalamus and Physiological Parameter Collection

A total of 110 male offspring voles and 54 paternal voles were sacrificed and dissected at different sampling times as mentioned above. The voles were anesthetized with ether, weighed, and then decapitated for trunk blood collection. Hypothalamic tissue was harvested, and the mass of testes and seminal vesicles were measured.

Hypothalamus sampling was restricted to the morning hours between 9:00 and 12:00 to mitigate the influence of daily fluctuations on gene expression. After decapitation, vole brains were expeditiously excised and the hypothalamus was dissected following the protocol outlined by Prendergast et al. (2013) [22], with demarcations at the optic chiasm anteriorly, the mammillary bodies posteriorly, and the hypothalamic sulci laterally. Following dissection, hypothalamus samples were frozen on dry ice and maintained at −80 °C for subsequent RNA isolation.

Trunk blood samples were centrifuged at 10,000 rpm for 3 min. Serum aliquots were then stored in polypropylene microcentrifuge tubes at −20 °C for testosterone radioimmunoassay. Testosterone levels in all serum samples were quantified using a single radioimmunoassay (RIA) by ^125^I RIA kits (Kemei Institute of Biotechnology, Beijing, China). The human antiserum used is highly specific for hormones, with cross-reactivity with other steroid hormones being <0.01% and intra-assay variability being <10% for all samples.

### 2.4. RNA Isolation, cDNA Transcription, and Gene Expression Measurement

Total RNA was isolated using the Direct-zol^TM^ RNA MiniPrep kit (Zymo Research, Los Angeles, CA, USA) and its concentration was quantified with the NanoDrop 2000 spectrophotometer (Thermo Fisher Scientific, Carlsbad, NM, USA). The RNA samples exhibited satisfactory 260/280 ratios falling within the range of 1.8 to 2.0. RNA integrity was confirmed by 1.2% agarose gel electrophoresis, showing distinct bands in appropriate proportions. Subsequently, 400 ng of RNA was utilized to synthesize cDNA in a 20 μL reaction volume employing the One-Step gDNA Removal and cDNA Synthesis SuperMix kit (TransGen Biotech, Beijing, China).

Gene expression was measured following the methodology outlined in a previous study [21]. Primers for quantitative real-time PCR (qRT-PCR) were designed based on either fragments or full-length sequences of genes specific to Brandt’s vole, including *γ*-*actin* (OQ599899), *Dio2* (KX856007), *Dio3* (KX889114), *Kiss1* (KX833248), *Rfrp3* (KY038930), and *GnRH* (KY038929) (See Table 2). *γ-actin* was selected as the reference gene for its consistent expression across various photoperiod treatments and postnatal developmental stages.

*Dio2*, *Kiss1*, *Rfrp3*, and *GnRH* gene expressions were evaluated using the BioMark HD System (Fluidigm Sciences Inc., Sunnyvale, CA, USA). Initially, pooled primers at 50 nM concentrations were used for pre-amplification of cDNA with 14 cycles in a 5 μL volume, employing TaqMan PreAmp Master Mix (Applied Biosystems, Foster City, CA, USA). Subsequently, Exonuclease I was utilized for primer cleanup to eliminate unincorporated primers. A 1:14 dilution of the final products was prepared for qPCR reactions. Samples and assays were then loaded into the Dynamic Array IFC (Fluidigm Sciences Inc., Sunnyvale, CA, USA), and gene expression was quantified using SsoFast EvaGreen Supermix (Bio-Rad Laboratories, Inc., Hercules, CA, USA) with the Fluidigm Biomark HD system. For *Dio3*, which was undetectable in the BioMark HD System, relative expression was determined using SYBR Green PCR mix (Applied Biosystems, Foster City, CA, USA) on an Applied Biosystems 7500 (Applied Biosystems, Foster City, CA, USA). The thermal cycler protocol included an initial step at 94 °C for 5 min, followed by 40 cycles of 94 °C for 30 s, 60 °C for 30 s, 72 °C for 40 s. Three replicates were performed for each gene sample. Gene expression levels were normalized to *γ-actin* and analyzed using the 2^–ΔΔCT^ method.

### 2.5. Statistical Analysis

Two-way ANOVA was employed to assess the main effects of the photoperiod and postnatal week, as well as their interaction, on physiological parameters and gene expression. One-way ANOVA was utilized to evaluate differences among treatments at each time point. The effect size was reported using partial *η*^2^. Additionally, Fisher’s Least Significant Difference (LSD) test was conducted to determine the significance between pairs of groups at the same timepoint or between different time points within the same treatment group. To control for Type I errors due to multiple comparisons, the Benjamini and Hochberg False Discovery Rate (FDR) correction was applied to adjust the significance threshold. FDR correction was performed using the p.adjust() function in R. Statistical analyses were conducted using SPSS 19.0, and figures were generated using GraphPad Prism 9.0. The significance level was set at *α* = 0.05.

## 3. Results

### 3.1. Physiology

#### 3.1.1. Male Offspring

**Figure 2:**
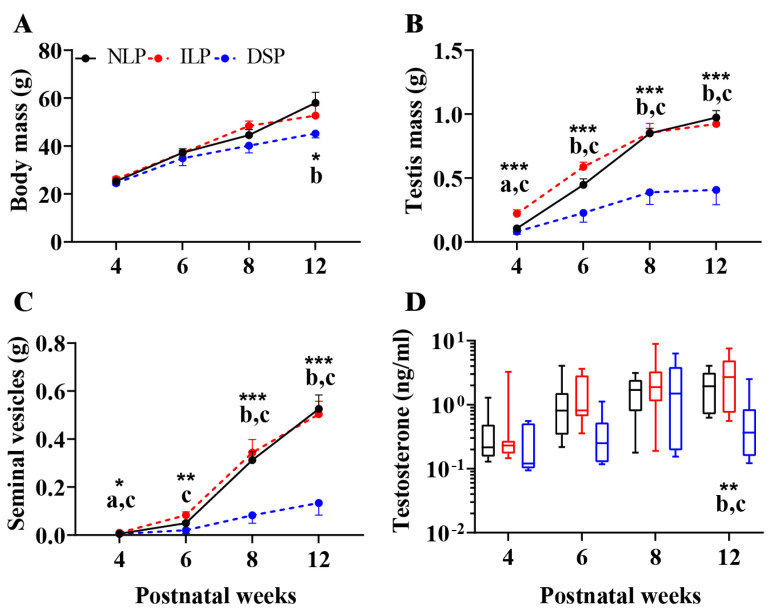
Physiological parameters of offspring voles under natural spring long photoperiod (NLP), increasing long photoperiod (ILP), and decreasing short photoperiod (DSP) during postnatal 12 weeks: (**A**) Body mass, (**B**) testis mass, (**C**) seminal vesicle mass, (**D**) serum testosterone levels of male offspring. Asterisks represent significant differences among the three groups; *: *p* < 0.05, **: *p* < 0.01, ***: *p* < 0.001. Above each timepoint, different signs indicate the significance among the three groups. Different letters indicate significant differences between groups: a, between NLP and ILP; b, between NLP and DSP; c, between ILP and DSP.

A two-way ANOVA for body mass revealed significant main effects of the photoperiod (*F*(2,98) = 6.192, *p* = 0.003, *η*^2^ = 0.112) and postnatal week (*F*(3,98) = 71.437, *p* < 0.001, *η*^2^ = 0.686), without an interaction between these factors (*F*(6,98) = 1.679, *p* = 0.134). At postnatal week 12, significant differences in body mass were found among the three groups (one-way ANOVA, *F*(2,23) = 3.803, *p* = 0.037, *η*^2^ = 0.248; Figure 2A). Post hoc analysis indicated that males in the NLP group had higher body mass compared to the DSP group (FDR = 0.036, 95% CI [−22.5087, −3.1424]; Figure 2A). Moreover, body mass increased significantly over time for males in all photoperiod groups: ILP (one-way ANOVA, *F*(3,29) = 32.020, *p* < 0.001, *η*^2^ = 0.768), DSP (*F*(3,34) = 16.179, *p* < 0.001, *η*^2^ = 0.588), and NLP (*F*(3,35) = 28.181, *p* < 0.001, *η*^2^ = 0.707).

Regarding testes mass, the analysis showed significant main effects of the photoperiod (*F*(2,98) = 45.908, *p* < 0.001, *η*^2^ = 0.484) and postnatal week (*F*(3,98) = 68.689, *p* < 0.001, *η*^2^ = 0.678), along with a significant interaction (*F*(6,98) = 4.993, *p* < 0.001, *η*^2^ = 0.234). Testes mass varied significantly across the three groups at postnatal week 4 (one-way ANOVA, *F*(2,27) = 10.982, *p* < 0.001, *η*^2^ = 0.449), 6 (*F*(2,24) = 10.312, *p* < 0.001, *η*^2^ = 0.462), 8 (*F*(2,24) = 14.193, *p* < 0.001, *η*^2^ = 0.542), and 12 (*F*(2,23) = 16.789, *p* < 0.001, *η*^2^ = 0.593; Figure 2B). Post hoc analysis showed that ILP males had higher testes mass than DSP males at postnatal week 4 (FDR < 0.001, 95% CI [0.0748, 0.2054]), 6 (FDR < 0.001, 95% CI [0.1939, 0.5257]), 8 (FDR < 0.001, 95% CI [0.2526, 0.6799]), and 12 (FDR < 0.001, 95% CI [−0.8275, −0.3606]). Additionally, NLP males exhibited greater testes mass than DSP males at postnatal week 6 (FDR = 0.012, 95% CI [−0.3763, −0.0626]), 8 (FDR < 0.001, 95% CI [−0.6632, −0.2591]), and 12 (FDR < 0.001, 95% CI [0.2939, 0.7947]). ILP males also had heavier testes than NLP males at postnatal week 4 (FDR = 0.002, 95% CI [0.049, 0.1796]). Testes mass increased significantly over time for males in all photoperiod groups: ILP (one-way ANOVA, *F*(3,29) = 45.631, *p* < 0.001, *η*^2^ = 0.825), DSP (*F*(3,34) = 3.313, *p* = 0.031, *η*^2^ = 0.226), and NLP (*F*(3,35) = 90.999, *p* < 0.001, *η*^2^ = 0.886).

For seminal vesicle mass, a two-way ANOVA indicated significant main effects of the photoperiod (*F*(2,97) = 36.307, *p* < 0.001, *η*^2^ = 0.428) and postnatal week (*F*(3,97) = 87.323, *p* < 0.001, *η*^2^ = 0.730), with a significant interaction (*F*(6,97) = 10.221, *p* < 0.001, *η*^2^ = 0.387). Significant differences were found at postnatal week 4 (one-way ANOVA, *F*(2,27) = 4.559, *p* = 0.020, *η*^2^ = 0.252), 6 (*F*(2,24) = 6.600, *p* = 0.005, *η*^2^ = 0.355), 8 (*F*(2,24) = 12.421, *p* < 0.001, *η*^2^ = 0.509) and 12 (*F*(2,22) = 17.096, *p* < 0.001, *η*^2^ = 0.608; Figure 2C). Post hoc analysis indicated that ILP males had higher seminal vesicles mass than DSP males at postnatal week 4 (FDR = 0.029, 95% CI [0.0011, 0.0079]), 6 (FDR = 0.004, 95% CI [0.0270, 0.0980]), 8 (FDR < 0.001, 95% CI [0.1406, 0.3830]), and 12 (FDR < 0.001, 95% CI [0.2057, 0.5345]), while NLP males had greater seminal vesicle mass than DSP males at postnatal week 8 (FDR < 0.001, 95% CI [−0.3451, −0.1159]) and 12 (FDR < 0.001, 95% CI [−0.5464, −0.2389]). Notably, ILP males had heavier seminal vesicles than NLP males at postnatal week 4 (FDR = 0.029, 95% CI [0.0007, 0.0075]). Additionally, seminal vesicle mass increased significantly over time for males in all groups: ILP (one-way ANOVA, *F*(3,29) = 40.950, *p* < 0.001, *η*^2^ = 0.809), DSP (*F*(3,33) = 3.931, *p* = 0.017, *η*^2^ = 0.263), and NLP (*F*(3,35) = 55.665, *p* < 0.001, *η*^2^ = 0.827).

For serum testosterone concentrations, a two-way ANOVA revealed significant main effects of the photoperiod (*F*(2,93) = 7.280, *p* = 0.001, *η*^2^ = 0.135) and postnatal week (*F*(3,93) = 13.538, *p* < 0.001, *η*^2^ = 0.304). At postnatal week 12, significant differences were observed among the three groups (one-way ANOVA, *F*(2,22) = 6.456, *p* = 0.006, *η*^2^ = 0.370; Figure 2D). Post hoc analysis indicated that ILP males had higher testosterone levels than DSP males (FDR = 0.009, 95% CI [0.1330, 0.5747]), and NLP males had higher testosterone levels than DSP males (FDR = 0.022, 95% CI [−0.4466, −0.0537]). Furthermore, testosterone levels varied significantly over time in all photoperiod groups: ILP (one-way ANOVA, *F*(3,28) = 5.005, *p* = 0.007, *η*^2^ = 0.349), DSP (*F*(3,30) = 3.681, *p* = 0.023, *η*^2^ = 0.269), and NLP (*F*(3,35) = 6.510, *p* = 0.001, *η*^2^ = 0.358).

#### 3.1.2. Paternal Voles

**Figure 3:**
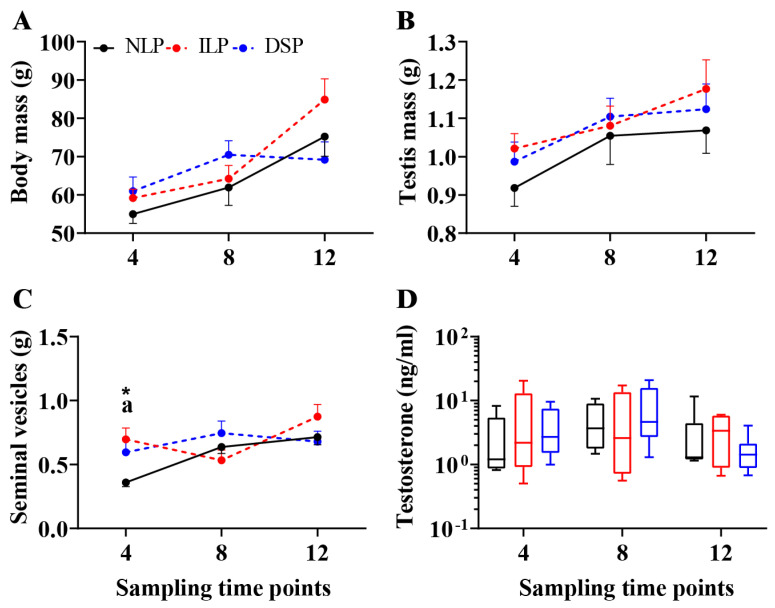
Physiological parameters of paternal voles under natural spring long photoperiod (NLP), increasing long photoperiod (ILP), and decreasing short photoperiod (DSP) during 12 weeks from offspring birth: (**A**) Body mass, (**B**) testis mass, (**C**) seminal vesicle mass, (**D**) serum testosterone levels of paternal voles. Asterisks represent significant differences among the three groups; *: *p* < 0.05. Above each timepoint, different signs indicate the significance among the three groups. Different letters indicate significant differences between groups: a, between NLP and ILP.

A significant main effect of the postnatal week was observed for body mass (two-way ANOVA, *F*(2,45) = 14.150, *p* < 0.001, *η*^2^ = 0.386). Paternal voles in both the ILP and NLP groups exhibited a significant increase in their body mass over time (one-way ANOVA, ILP: *F*(2,13) = 12.564, *p* = 0.001, *η*^2^ = 0.659; NLP: *F*(2,16) = 5.679, *p* = 0.014, *η*^2^ = 0.415; Figure 3A).

For testes mass, a significant main effect of the postnatal week was found (two-way ANOVA, *F*(2,45) = 5.025, *p* = 0.011, *η*^2^ = 0.183). However, no significant differences were observed among the three groups at any time point, nor were there significant developmental changes over time within each group (Figure 3B).

Significant main effects of the postnatal week were observed for seminal vesical mass (two-way ANOVA, *F*(2,45) = 4.845, *p* = 0.012, *η*^2^ = 0.177), with a significant interaction between the photoperiod and postnatal week (*F*(4,45) = 2.984, *p* = 0.029, *η*^2^ = 0.210). At postnatal week 4, significant differences were observed among the three groups (one-way ANOVA, *F*(2,15) = 4.258, *p* = 0.034, *η*^2^ = 0.362; Figure 3C). Post hoc analysis indicated that ILP males had heavier seminal vesicles than NLP males (FDR = 0.037, 95% CI [0.0846, 0.5904]). Furthermore, seminal vesicle mass increased significantly over time in the NLP condition (*F*(2,16) = 13.593, *p* < 0.001, *η*^2^ = 0.630).

No significant differences in serum testosterone levels were found based on either two-way or one-way ANOVA (Figure 3D).

### 3.2. Hypothalamic Gene Expression

#### 3.2.1. *Dio2*

**Figure 4:**
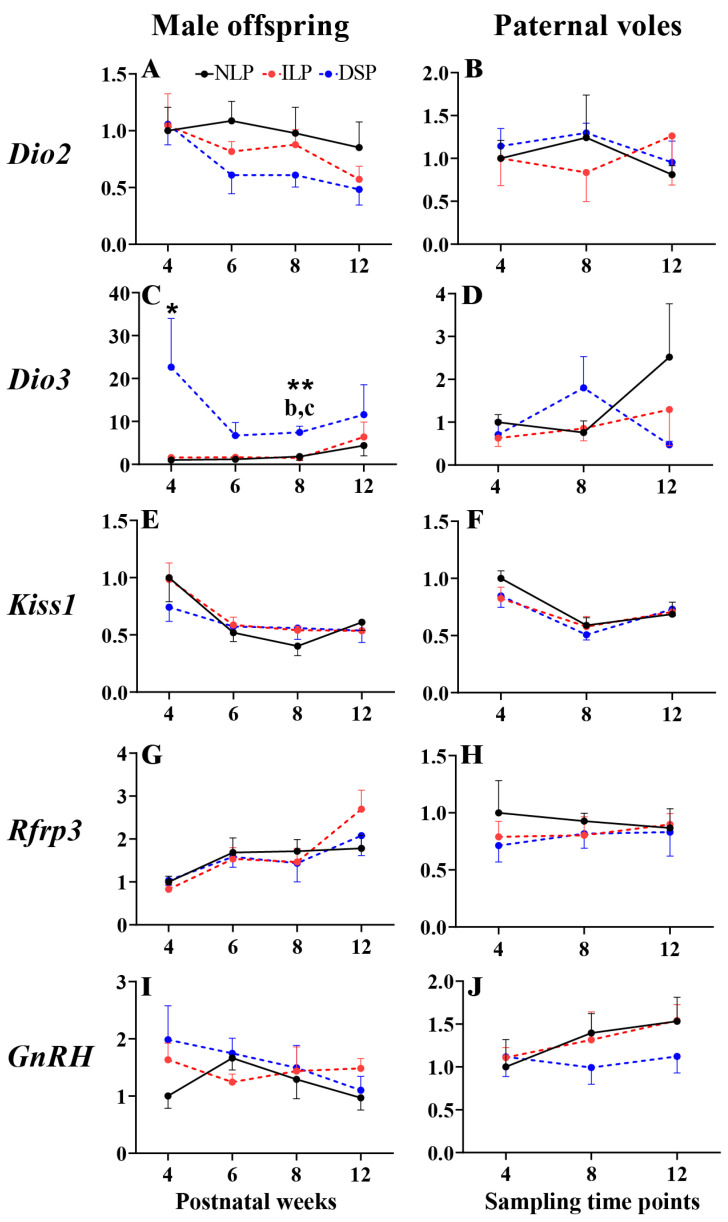
Hypothalamic gene expression of offspring and paternal voles under natural spring long photoperiod (NLP), increasing long photoperiod (ILP), and decreasing short photoperiod (DSP) during 12 weeks from offspring birth. Relative expression of (**A**,**B**) *Dio2*, (**C**,**D**) *Dio3*, (**E**,**F**) *Kiss1*, (**G**,**H**) *Rfrp3*, (**I**,**J**) *GnRH* in the hypothalamus from male offspring and paternal voles, respectively. Asterisks represent significant differences among the three groups; *: *p* ≤ 0.05, **: *p* < 0.01. Above each timepoint, different signs indicate the significance among the three groups. Different letters indicate significant differences between groups: b, between NLP and DSP; c, between ILP and DSP.

Two-way ANOVA did not reveal any main effects of the photoperiod or postnatal week, nor an interaction between the two, on hypothalamic *Dio2* expression in offspring and paternal voles. Developmental changes were not evident in either the offspring or paternal groups (Figure 4A,B).

#### 3.2.2. *Dio3*

A significant main effect of the photoperiod on hypothalamic *Dio3* expression in male offspring was observed (two-way ANOVA, *F*(2,78) = 7.763, *p* = 0.001, *η*^2^ = 0.166). Significant variations were found across groups at postnatal week 4 (one-way ANOVA, *F*(2,18) = 3.558, *p* = 0.050, *η*^2^ = 0.283) and 8 (*F*(2,21) = 11.337, *p* < 0.001, *η*^2^ = 0.519). At postnatal week 4 and 6, DSP male offspring showed a near-significant increase compared to both NLP and ILP groups (FDR = 0.064 and FDR = 0.086, respectively). At postnatal week 8, DSP strongly stimulated *Dio3* expression in male offspring compared to the ILP group (FDR < 0.001, 95% CI [−8.838364, −3.014871]; Figure 4C) and NLP group (FDR < 0.001, 95% CI [−8.519607, −2.696113]; Figure 4C). No photoperiodic and developmental effect was detected in the paternal groups (Figure 4D).

#### 3.2.3. *Kiss1*

A significant main effect of the postnatal week on hypothalamic *Kiss1* expression was observed in both male offspring (two-way ANOVA, *F*(3,76) = 8.989, *p* < 0.001, *η*^2^ = 0.262) and paternal voles (*F*(2,38) = 13.738, *p* < 0.001, *η*^2^ = 0.420). With the development, significant decreases in expression were found in the two LP male offspring groups (one-way ANOVA, NLP: *F*(3,25) = 4.073, *p* = 0.017, *η*^2^ = 0.328; ILP: *F*(3,25) = 6.181, *p* = 0.003, *η*^2^ = 0.426; Figure 4E), as well as in the NLP (*F*(2,12) = 6.887, *p* = 0.010, *η*^2^ = 0.534) and DSP (*F*(2,14) = 6.438, *p* = 0.010, *η*^2^ = 0.479) paternal groups (Figure 4F). No photoperiodic treatment effect was detected in any of the groups.

#### 3.2.4. *Rfrp3*

A significant main effect of the postnatal week on hypothalamic *Rfrp3* expression was observed in male offspring (two-way ANOVA, *F*(3,77) = 7.594, *p* < 0.001, *η*^2^ = 0.228). Developmental changes indicated notable increases in expression in the ILP male offspring group (one-way ANOVA, *F*(3,26) = 6.073, *p* = 0.003, *η*^2^ = 0.412). No photoperiodic treatment effect was evident in any of the groups (Figure 4G,H).

#### 3.2.5. *GnRH*

No photoperiodic or developmental effects were found in either the male offspring or paternal groups (Figure 4I,J).

## 4. Discussion

### 4.1. More Similar Photoperiodic Pattern Produced Closer Physiological and Molecular Responses

This study reveals that male offspring in the NLP and ILP groups exhibited similar physiological development and gene expression patterns compared to those in the DSP group. Specifically, the NLP and ILP groups demonstrated increased body and gonadal masses, decreased hypothalamic *Dio3* expression, and elevated testosterone levels. These results indicate that both the artificial photoperiod schedule (increasing light duration by 3 min per day) and the natural, gradually increasing photoperiod yield comparable outcomes in gonadal development and gene expression in Brandt’s voles.

Reports on the effects of varying photoperiod lengths have been documented. For instance, field voles (*Microtus agrestis*) showed minimal growth rate under 13 h of light, while 13.5 h or more led to progressively heavier testes [46]. Siberian hamsters exhibited rapid gonadal development before 6 weeks post-summer solstice, which slowed thereafter [47]. Common voles (*Microtus arvalis*) display a gradient change in testicular activity and hypothalamic *Dio2* and *Dio3* expression from 16L:8D to 6L:18D in two-hour intervals [48]. Despite only using two patterns of LP, similarities were observed between the two groups, with significant differences from the DSP group. This study is the first to compare photoperiodic responses across various gradually changing patterns. Notably, differences were found between ILP and NLP at postnatal week 4, particularly in testes and seminal vesicle mass of male offspring, indicating sensitivity to subtle photoperiod changes near weaning. These findings highlight the importance of considering early subtle photoperiod differences in developmental responses. This insight is valuable for designing future study photoperiod settings.

### 4.2. Divergent Photoperiodic Responses Between Male Offspring and Paternal Voles

An intriguing finding of our study is that male offspring exhibited distinct gonadal activity and hypothalamic *Dio3* expression under LP and SP conditions, while paternal voles did not show a significant response to these treatments. Additionally, our field research demonstrated that adult male voles, in comparison to newborn juveniles, exhibited continuous gonadal development and elevated testosterone levels in early autumn after overwintering, whereas newborn males experienced complete gonadal suppression [21]. Monitoring fecal testosterone levels in semi-natural enclosures, indicated that gonadal inhibition commenced around the summer solstice [44].

Despite being exposed to DSP, male offspring still exhibited a significant development of gonad glands during the 12-week treatment, indicating a reduced inhibitory effect of SP on gonadal gland development. This phenomenon may be linked to photorefractoriness, as seen in hamsters exposed to SP for over 10 weeks [49,50]. The hypothalamic *Dio3* gene likely plays a pivotal role in this process. Previous studies in Syrian or Siberian hamsters demonstrated that gonadal glands that were initially suppressed under SP exposure began to reactivate after 12 or 16 weeks, respectively. Additionally, the expression of hypothalamic *Dio3* decreased significantly compared to *Dio2* gene expression [6]. Data also revealed a notable decrease in hypothalamic *Dio3* expression in 12-week-old male offspring exposed to DSP compared to those at 4 weeks of age. These findings suggest that maintaining high levels of hypothalamic *Dio3* expression is essential for responding to SP.

Our study provides initial evidence on the comparison of hypothalamic *Dio2* and *Dio3* expression levels in old and young rodents. In male offspring exposed to the DSP condition, a significant decrease in hypothalamic *Dio3* expression was observed, while in paternal voles, no significant difference was found in gonadal mass or hypothalamic *Dio2* and *Dio3* expression. These results support the presence of photoperiodism in juvenile Brandt’s voles, partially explaining the inability of the SP condition to inhibit gonadal activity in wild voles as noted in our previous study [21].

In terms of ecological significance, paternal voles display reduced sensitivity to photoperiodic changes, possibly attributed to their short lifespan in the wild, typically lasting only several months. Surviving overwintered males resume gonadal function before the breeding season begins, playing a crucial role in driving breeding efforts throughout the reproductive season. Given their short lifespan, often not exceeding a second winter, it is essential for these males to maximize reproductive output to ensure species survival.

### 4.3. Hypothalamic Dio3 Probably Plays an Important Role in Regulating the Seasonal Breeding of Brandt’s Vole

Compared to the NLP and ILP groups, DSP treatment significantly inhibited somatic and gonadal development in male offspring, accompanied by markedly increased hypothalamic *Dio3* expression, particularly in early stages, while showing only a slight inhibitory effect on *Dio2* expression. These findings indicate a potentially pivotal role of *Dio3* in regulating local T_3_ levels in the hypothalamus, thereby transmitting photoperiodic signals to modulate seasonal responses in young Brandt’s voles. The modulation of local T_3_ concentration stimulates GnRH activity in the hypothalamus, facilitating gonadal growth in avian and rodent species [27,51,52,53]. Previous studies have identified hypothalamic *Dio2* as the key photoperiodic gene involved in seasonal transitions in Japanese quail (*Coturnix coturnix japonica*) [51], Siberian hamster [54], and Syrian hamster [55]. Subsequently, the *Dio3* gene was discovered, exhibiting an opposing photoperiodic response to *Dio2* [22,56]. In this study, *Dio3* exhibited higher expression in DSP offspring at postnatal week 4. While data prior to postnatal week 4 were not available, hindering the determination of expression patterns before this age, previous research using in situ hybridization in Siberian hamsters revealed that hypothalamic *Dio2* displayed elevated expression under LP condition at birth, whereas *Dio3* showed no difference at birth but exhibited higher expression under SP condition at postnatal day 15, when *Dio2* expression no longer differed significantly [10]. Whether Brandt’s voles exhibit similar expression patterns during early postnatal development warrants further investigation.

However, in line with our findings, previous studies have indicated a relatively weaker photoperiodic sensitivity of the *Dio2* gene compared to the *Dio3* gene. For example, research on Siberian hamsters demonstrated that changes in *Dio3* expression, rather than *Dio2*, were notably affected by various photoperiodic and T_3_ treatments. These changes were observed through semiquantitative in situ hybridization in adult hamsters [52] and quantitative qPCR in juvenile hamsters [22]. Shifting photoperiod treatments between LP and SP conditions resulted in significant alterations in hypothalamic *Dio3* expression in both male and female hamsters, whereas changes in *Dio2* expression were only observed in males [57]. Notably, under SP conditions and melatonin treatment, only hypothalamic *Dio3* expression showed a significant increase, with no corresponding change in *Dio2* expression [58]. Furthermore, our field study on Brandt’s voles revealed that the seasonal fluctuation of hypothalamic *Dio2* expression was less pronounced compared to *Dio3* expression [21]. Additionally, *Dio3* expression consistently decreased with gonadal development in male offspring, suggesting a weakening of its inhibitory effects on the gonadal gland. These findings collectively suggest that the hypothalamic *Dio3* gene likely plays a more pivotal role in regulating local T_3_ concentration during seasonal transitions in newborn Brandt’s voles. This conclusion is reinforced by a study on tundra voles (*Microtus oeconomus*) and common voles, which highlighted the significant influence of the photoperiod on *Dio2* and *Dio3* expression in the developing hypothalamus of both species [59]. Additionally, *Dio2* expression was found to be highly responsive to changes in ambient temperature, particularly in the spring-programmed tundra voles, while *Dio3* expression exhibited greater sensitivity to variations in the photoperiod [60].

Surprisingly, *Kiss1* and *Rfrp3* were found to be essential for puberty onset and reproductive activation in mammals [23,25,61,62,63], as well as for responding to the photoperiod and melatonin [25,28,64]. However, our current data contradict these established roles, as we did not observe any photoperiodic differences in the expression of these genes, consistent with another study [45]. It is possible that the expression patterns of these genes have a complex temporal dynamic during the development of young rodents [65,66]. Taken together, these findings highlight the significant role of *Dio3* in mediating seasonal physiological adaptations influenced by the photoperiod.

This study was limited by the absence of sampling before postnatal week 4, preventing the assessment of early developmental changes and gene expression patterns. The existence and significance of early differences in gene expression remain unclear. This limitation may have led to an overemphasis on *Dio3*’s role, potentially overlooking early-stage effects of other genes. Additionally, the lack of continuity in tracking individuals from juvenile to adult stages hindered the observation of longitudinal changes within subjects. The random sampling of different individuals at each time point may have introduced individual variations, complicating the interpretation of changes in gonadal responses to age-related photoperiodic changes. Future studies could explore these limitations by incorporating earlier timepoint samples and monitoring individual development across various life stages.

## 5. Conclusions

In summary, we found that exposure to DSP impeded the gonadal development of juvenile Brandt’s voles and led to a distinct difference in hypothalamic *Dio3* expression compared to ILP- and NLP-exposed voles. Adult male voles exhibited decreased sensitivity to short photoperiods, as indicated by alterations in the gonadal gland and hypothalamic *Dio3* expression. These findings imply that hypothalamic *Dio3* likely plays a pivotal role in modulating local T_3_ concentration to regulate the developmental trajectory of the gonadal gland in Brandt’s voles.

## Figures and Tables

**Table 1:** Sampling numbers for each group across different postnatal timepoints.

Postnatal Week	Male Offspring (Number)			Paternal Voles (Number)		
	ILP	DSP	NLP	ILP	DSP	NLP
4 weeks	10	10	10	6	6	6
6 weeks	8	9	10	-	-	-
8 weeks	8	9	10	6	6	6
12 weeks	7	10	9	7	7	4

Note: ILP, increasing long photoperiod; DSP, decreasing short photoperiod; NLP, natural increasing long photoperiod.

**Table 2:** Primers sequences of Brandt’s vole genes.

Genes	Length	Annealing Temperature	Primer Sequence
*γ-actin*	113 bp	62 °C	F: GCTCTCTTCCAGCCTTCCTTCCTG R: GTGTTGGCGTACAGGTCCTTGCGG
*Dio2*	103 bp	62 °C	F: TGCCTACAAACAGGTTAAATTGGGT R: GGCTGTCTTCTTCAAGGCATAA
*Dio3*	136 bp	62 °C	F: TCAACAGTGAAGGCGAGGAGGT R: TCGTGGGCCTGCTTGAAGAAAT
*GnRH*	124 bp	62 °C	F: CGATTCTTTCCAAGAGATGGG R: CATCAGACTTTCCAGAGCTCCT
*Kiss1*	143 bp	62 °C	F: CACTGGCTTCTTGGCAGCTACTG R: GCCCTTTTCCCAGGCATTGA
*Rfrp3*	112 bp	62 °C	F: GACAAATATCTCCAGCCTAGAGG R: GGGCTGGACTCATCTTAATAACAT

## Data Availability

The original contributions presented in this study are included in the article. Further inquiries can be directed to the corresponding authors.
